# A decoupled alignment kernel for peptide membrane permeability predictions

**DOI:** 10.1186/s13321-026-01276-5

**Published:** 2026-08-04

**Authors:** Ali Amirahmadi, Gökçe Geylan, Leonardo De Maria, Farzaneh Etminani, Mattias Ohlsson, Alessandro Tibo

**Affiliations:** 1 Center for Applied Intelligent Systems Research in Health, Halmstad University, Kristian IV:s väg 3, 30118 Halmstad, Sweden; 2 Division of Systems and Synthetic Biology, Department of Life Sciences, Chalmers University of Technology, Kemigården 1, 41296 Gothenburg, Sweden; 3 Molecular AI, Discovery Sciences, R&D, AstraZeneca, Pepparedsleden 1, 43183 Mölndal, Sweden; 4 Medicinal Chemistry, Research and Early Development, Respiratory & Immunology, BioPharmaceuticals R&D, AstraZeneca, Pepparedsleden 1, 43183 Mölndal, Sweden; 5 Department of Research and Development (FoU), Region Halland, Södra vägen 9, 30118 Halmstad, Sweden; 6 Centre for Environmental and Climate Science, Computational Science for Health and Environment, Lund University, Kontaktvägen 10, 22362 Lund, Sweden

**Keywords:** Cyclic peptides, Permeability, Gaussian processes, Global alignment kernel, Tanimoto, Calibration

## Abstract

Cyclic peptides are promising modalities for targeting intracellular sites; however, cell-membrane permeability remains a key bottleneck, exacerbated by limited public data and the need for well-calibrated uncertainty. Instead of relying on data-eager complex deep learning architecture, we propose a monomer-aware decoupled global alignment kernel (MD-GAK), which couples chemically meaningful residue–residue similarity with sequence alignment while decoupling local matches from gap penalties. MD-GAK is a relatively simple kernel. To further demonstrate the robustness of our framework, we also introduce a variant, PMD-GAK, which incorporates a triangular positional prior. As we will show in the experimental section, PMD-GAK can offer additional advantages over MD-GAK, particularly in reducing calibration errors. Since our focus is on uncertainty estimation, we use Gaussian Processes as the predictive model, as both MD-GAK and PMD-GAK can be directly applied within this framework. We demonstrate the effectiveness of our methods through an extensive set of experiments, comparing our fully reproducible approach against state-of-the-art models, and show that it outperforms them across all metrics.

**Scientific contribution**

We introduce monomer-aware decoupled global alignment kernels for Gaussian processes (MD-GAK and position-aware PMD-GAK) that align cyclic peptides at the sequence level using chemically rich monomer fingerprints and explicit positional priors, yielding positive-definite similarity measures tailored to permeability modeling. Compared with order-agnostic fingerprint methods, standard global-alignment kernels and state-of-the-art graph and language-model baselines, our alignment-aware GPs provide improved discrimination, probabilistic calibration and scaffold-level robustness under stringent, leakage-controlled cyclic-peptide permeability benchmarks.

**Supplementary Information:**

The online version contains supplementary material available at 10.1186/s13321-026-01276-5.

## Introduction

Cyclic peptides have re-emerged as a compelling modality for intracellular targets thanks to their high affinity and selectivity, yet their *cell membrane permeability* remains a central bottleneck for discovery [1, 2]. Recent resources such as Cyclic Peptide Membrane Permeability Database (CycPeptMPDB) [1] aggregate permeability measurements from the literature and patents across multiple assays (notably Parallel artificial membrane permeability assay (PAMPA)), providing a large and well-annotated dataset for systematic modeling [1]. However, these datasets are still relatively small by modern machine learning (ML) standards, heterogeneous in experimental provenance, and structurally biased, which complicates out-of-domain generalization and model calibration [2, 3]. These challenges underscore the need for methods that can (i) encode peptide-specific structure, (ii) work well in the small–to–medium data regime, and (iii) provide reliable uncertainty quantification.

Gaussian processes (GPs) are natural candidates for such settings: they deliver calibrated probabilistic predictions with an inductive bias entirely controlled by a *kernel* [4, 5]. The kernel choice is therefore pivotal. For small molecules, the Tanimoto (Jaccard) kernel on circular fingerprints (e.g., RDKit Morgan) has been highly successful. These fingerprints encode rich local topological environments via circular neighborhoods. However, when a cyclic peptide is represented as a single molecular graph, circular fingerprints are inherently order-agnostic with respect to the peptide’s monomer sequence [6, 7]: they encode local topological environments but do not preserve the explicit residue order or enable residue-to-residue alignment. Since permeability depends not only on which building blocks are present but also on where they occur and which neighbors they have [8, 9], this motivates kernels that pair chemistry-aware monomer similarities with sequence alignment, capturing both composition and arrangement.

Recent benchmarking on cyclic peptide permeability underscores that representation and split strategy (e.g., scaffold versus random splits) can dominate performance and robustness trends, and that calibrated uncertainty is as important as raw discrimination [3]. The work of parallel methodology also emphasizes careful data curation, duplicate control, and applicability domain-aware evaluation to avoid optimistic estimates under nearly duplicate leakage [2].

To retain peptide-specific structure, one can move beyond features from traditional fingerprint encodings that summarize each peptide as an order-invariant collection of local substructures and instead employ sequence- and graph-aware representations. Circular fingerprints such as Morgan/ECFP encode rich local chemistry and remain strong baselines for small-molecule QSAR; however, when a single global fingerprint is used as the only feature for a peptide, structural information (e.g. order and long-range topology) [10], and subtle stereochemical rearrangements [11] are largely missed. As a result, traditional classifiers such as SVMs or random forests often show limited discriminative power for cyclic peptide permeability when compared with modern graph-based models [3]. On the sequence side, a large body of work now treats molecules explicitly as strings or token sequences. Recurrent and transformer-based chemical language models (e.g., LSTMs, ChemBERTa [12], and PeptideCLM [13]) operate on SMILES and have demonstrated that sequence-aware encoders can capture both local and more global structural patterns that are not easily accessible to a single fingerprint vector [12]. Within kernel methods, *Global Alignment* (GA) kernels provide a principled way to exploit sequence order: they replace the hard minimum of Dynamic Time Warping (DTW) [14] with a soft sum over all monotone alignments, yielding positive-definite similarities suitable for kernel machines and Gaussian processes [15]. "Triangular" GA variants introduce a kernel that both encodes positional priors and reduces computation, maintaining positive definiteness [4].

Graph-aware methods push this idea further by working directly on the molecular graph. Message-Passing Neural Networks (MPNNs) unify a broad family of graph convolutional architectures into a common message-passing framework, achieving state-of-the-art performance on several quantum-chemical benchmarks by learning task-specific node and edge embeddings from molecular graphs [16]. AttentiveFP builds on this paradigm by introducing a graph attention mechanism that adaptively reweights neighbors: it not only propagates information along local bonds but also learns nonlocal intramolecular interactions and “hidden” edges that are most relevant for a given property, while still respecting the underlying molecular topology [17]. Directed MPNNs (DMPNNs) refine message passing even further by operating on directed edges and improve information flow [18]. Recent systematic benchmarking on cyclic peptide membrane permeability demonstrates that graph-based models such as AttentiveFP, MPNN, and DMPNN are among the top-performing approaches, clearly outperforming classical SVM and random-forest models built solely on fingerprint descriptors [3].

In this work, we take a complementary approach that combines the strengths of rich local chemistry of molecular fingerprints with sequence-aware topology. We introduce a *monomer decoupled global alignment kernel* (MD-GAK) for cyclic peptides. Each peptide is represented as an ordered sequence of monomer units. Our key design choice is to treat each monomer as a small molecule and encode it with a Morgan fingerprint, while explicitly retaining both the sequence order and the chirality of the monomers. Chirality is critical for Peptide permeability as minimal changes—as simple as a single stereochemical inversion—can rewire intramolecular networks and conformational ensembles and thus shift membrane permeability [1, 8, 9]. By feeding the resulting sequence of monomer-level fingerprints into a GA kernel, MD-GAK captures sequential topology while leveraging chemically rich local descriptors, yielding a structured similarity measure that is directly usable within kernel machines and Gaussian process models for cyclic peptide permeability prediction.

While we consider simple kernels on monomers in this work, our approach will, in principle, allow us to use any complex kernel (e.g., based on neural networks, transformers,...) for small molecules for which we have access to billions of data points, in contrast to peptides, where the number of structures is limited to a few thousand. This decoupling lets us compare peptides via *monomer–monomer* chemistry—where small-molecule tooling, fingerprints, and kernels are mature. We score local matches using a Tanimoto kernel on monomer fingerprints and then aggregate over all residue alignments with a GA-style dynamic program to form the peptide–peptide kernel. We further propose a *position-aware* variant (PMD-GAK) that adds a triangular positional prior, reducing computational cost and discouraging implausible warpings (insertions and deletions in alignment). Embedded in a GP classifier, these kernels combine chemically grounded local similarity with flexible sequence alignment and yield calibrated predictive uncertainties—leveraging the strengths of small-molecule representations while capturing the sequence organization unique to peptides.

*Empirical scope.* Using CycPeptMPDB, we assess our approach in two complementary regimes: (i) an applicability-domain–aware nested cross-validation with label- and canonical-group–stratified folds to curb leakage and probe robustness [2]; and (ii) a length-focused subset (6-, 7-, and 10-mers) evaluated under random and scaffold splits, following recent benchmarks [3]. Across both settings we compare against strong baselines. We observe consistent gains in discrimination (ACC/F1/ROC–AUC) and improved probabilistic calibration (Brier/ECE), with the position-aware variant particularly enhancing calibration.

*Contributions.* (1) A peptide-specific *monomer decoupled global alignment Kernel* that aligns sequences of monomer-level molecular fingerprints, bridging local chemical similarity and sequence order. (2) A *position-aware* triangular variant (PMD-GAK) that encodes positional priors and improves calibration while retaining positive-definite structure. (3) A thorough evaluation on CycPeptMPDB under leakage-aware protocols and length-focused splits, showing that alignment-aware GPs exceed strong baselines and yield improved probabilistic calibration. (4) A theoretical construction (Appendix.1) proving the positive semidefiniteness of our kernel via a rational/convolution kernel argument, and a discussion of its relation to classical GA/Triangular-GA kernels and modern graph GP kernels [5].

Overall, our results indicate that *monomer-aware alignment* is a practical and principled route to modeling cyclic peptide permeability: it captures sequence-sensitive chemistry, behaves well in the low-data regime typical of permeability measurements, and integrates seamlessly with GP-based uncertainty quantification—a combination that directly addresses the reliability concerns raised in recent methodological studies [2, 3].

## Methods

We cast peptide–peptide comparison as a positive-definite global-alignment kernel at the monomer level and use it as the covariance of a Gaussian process (GP). This connects chemically meaningful local similarity with sequence order, while retaining closed-form GP inference and calibrated uncertainties.

### Gaussian process

A Gaussian process (GP) is a distribution over functions $f:\mathbb {R}^n\!\rightarrow \!\mathbb {R}$ such that any finite collection has a joint Gaussian distribution:

$$f(\cdot )\sim \mathcal{G}\mathcal{P}\big (m(\cdot ),\,k(\cdot ,\cdot )\big ),$$

with mean function m and positive-definite covariance (kernel) k. For a dataset $\mathcal {D} = \{ (x_i \in \mathbb {R}^n, y_i\in \mathbb {R})\}_{i=1}^{N}$, we represent all the inputs in a matrix *X* of size N×n and the outputs in a vector $\textbf{y}$ of size *N*. The induced prior over function values is

$$\textbf{f}:=f(X)\sim \mathcal {N}\!\big (m(X),\,K_{XX}\big ),\quad (K_{XX})_{ij}=k(x_i,x_j).$$

In regression tasks, we assume that the labels are noisy, i.e. $y_i = f(x_i) + \varepsilon _i$ with $\varepsilon _i \sim \mathcal {N}(0,\eta ^2)$, where η>0 denotes the observation noise standard deviation.

*Joint prior over train/test (regression).* Let $X_*$ denote test inputs and $\textbf{f}_*:=f(X_*)$. The joint prior over observed targets $\textbf{y}$ and test function values is

$$\begin{bmatrix} \textbf{y}\\ \textbf{f}_* \end{bmatrix} \sim \mathcal {N}\!\left( \begin{bmatrix} m(X)\\ m(X_*) \end{bmatrix}, \begin{bmatrix} K_{XX}+\eta ^2 I & K_{X X_*}\\ K_{X_* X} & K_{X_* X_*} \end{bmatrix} \right) ,$$

with $$K_{X X_*}=k(X,X_*)$$, $$K_{X_* X}=K_{X X_*}^{\top }$$, and $$K_{X_* X_*}=k(X_*,X_*)$$.

*Posterior (regression).* Conditioning the GP on $(X,\textbf{y})$, we obtain a Gaussian posterior for $\textbf{f}_*$:

$$\begin{aligned} & \bar{m}_* \;=\; m(X_*) \;+\; K_{X_* X}\big (K_{XX}+\eta ^2 I\big )^{-1}\!\big (\textbf{y}-m(X)\big ),\\ & \bar{\Sigma }_* \;=\; K_{X_* X_*} \;-\; K_{X_* X}\big (K_{XX}+\eta ^2 I\big )^{-1} K_{X X_*}. \end{aligned}$$

**Fig. 1:**
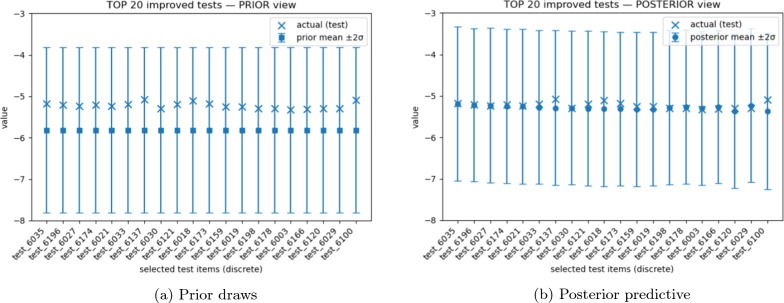
GP prior and posterior for PAMPA prediction using the proposed kernel. The data conditioning contracts the posterior uncertainty and shifts the mean toward test samples observations

The posterior mean provides point predictions and $\bar{\Sigma }_*$ quantifies predictive uncertainty (Fig. 1).

*Binary GP classification (Laplace).* For binary labels $$y_i\in \{0,1\}$$ we place a zero-mean GP prior on latent logits, $$\textbf{f}\sim \mathcal {N}(0,K)$$, and use a logistic likelihood $$p(y_i=1\mid f_i)=\Psi (f_i)$$ with $$\Psi (z)=1/(1+e^{-z})$$. The approximate posterior over $$\textbf{f}$$ is $$\mathcal {N}(\hat{\textbf{f}},\,\Sigma )$$ with $$\Sigma =(K^{-1}+W)^{-1}$$, where $$\hat{\textbf{f}}$$ is obtained using a Laplace approximation [19, 20] and $$W=\textrm{diag}\!\big (\Psi (\hat{\textbf{f}})\odot (1-\Psi (\hat{\textbf{f}}))\big )$$. For a test input $$x_*$$ with $$k_*:=k(X,x_*)$$ and $$k_{**}:=k(x_*,x_*)$$,

$$\begin{aligned} & \mu _{f_*}=k_*^{\top }\,K^{-1}\,\hat{\textbf{f}}, \quad \sigma _{f_*}^2=k_{**}\;-\;k_*^{\top }\,\big (K+W^{-1}\big )^{-1}k_*,\\ & p(y_*=1 \, | \, X, y, x_*) = \int \sigma (f_*)\,\mathcal {N}(f_*;\mu _{f_*},\sigma _{f_*}^2)\,df_*. \end{aligned}$$

#### Kernel methods

In Gaussian processes (GPs), specifying a covariance (kernel) k determines a prior over functions and thereby encodes the inductive bias of the model. The predictive behavior of a GP depends critically on this choice: different kernels express different notions of similarity (e.g. smoothness, invariance, compositional structure) and thus suit different input types (e.g. strings, graphs, molecules).

Kernel methods specify a positive-definite kernel $k(x,x^{\prime })$ that represents an inner product in a (possibly infinite-dimensional) feature space $$\mathcal {H}$$, i.e.

$$k(x,x') \;=\; \langle \phi (x), \phi (x') \rangle _{\mathcal {H}}$$

for some (typically implicit) feature map ϕ. A positive definite kernel $k:\mathcal {X}\times \mathcal {X}\rightarrow \mathbb {R}$ on an input space $\mathcal {X}$ implicitly defines an RKHS $\mathcal {H}_k$ in which linear algorithms operate via Gram matrices $K_{ij}=k(x_i,x_j)$ (the “kernel trick”) [21, 22]. In GPs, the kernel plays the role of a covariance function that encodes prior smoothness and inductive bias; posterior predictions follow in closed form given *K* and a likelihood [20]. Consequently, the modelling power of a GP hinges on choosing a kernel tailored to the structure of the inputs (e.g. strings, graphs, molecules), rather than generic Euclidean distances.

### Monomer global alignment kernel

#### Global alignment

Given two sequences $A=(a_1,\ldots ,a_n)$ and $B=(b_1,\ldots ,b_m)$ of lengths *n* and *m*, respectively over an alphabet $\mathcal {A}$, a (global) alignment is a pair of nondecreasing index paths $(\pi _1,\pi _2)$ with unit steps (1, 0), (0, 1), or (1, 1) that map *A* to *B* through substitutions and gaps. Dynamic programming (DP) aggregates local substitution scores and gap operations from boundary conditions to the terminal cell, in *O*(*nm*) time [4].

While the Dynamic Time Warping (DTW) distance is a de-facto baseline for sequence comparison, it is *not* a metric and is known to be not negative definite [4]; consequently, kernels derived directly from DTW (e.g., $\exp (-\gamma \,\textrm{DTW})$) are not guaranteed positive definite and can break RKHS-based learning. Global Alignment (GA) kernels address this by replacing the hard minimum in DTW with a sum over all monotone alignments (a soft-min), yielding a similarity that can be made positive definite under mild conditions on the local kernel and gap weighting [4, 15]. GA kernels retain DTW’s *O*(*nm*) complexity but, unlike DTW’s single best path, they summarize the entire ensemble of alignments costs, which is often more informative for learning [4, §2.2].

#### Global alignment kernels

Let $\kappa :\mathcal {X}\times \mathcal {X}\rightarrow \mathbb {R}_{\ge 0}$ be a local similarity (typically a valid kernel on tokens; e.g., Tanimoto on fingerprints or any PSD similarity defined on monomers). The Global Alignment (GA) kernel sums contributions over all alignments and can be computed by the DP recursion

1 $$\begin{aligned} \begin{aligned} M_{0,0}&=1,\qquad M_{i,0}=0,\ M_{0,j}=0,\quad i\ge 1,\ j\ge 1,\\ M_{i,j}&=\kappa (a_i,b_j)\,\big (M_{i-1,j-1}+M_{i-1,j}+M_{i,j-1}\big ), \end{aligned} \end{aligned}$$

and set $K_{\textrm{GA}}(A,B):=M_{n,m}$. Intuitively, each monotone path contributes the product of local similarities along its matched positions, while horizontal/vertical steps allow gaps. Positive definiteness is guaranteed if the transformed local kernel $\kappa /(1+\kappa )$ is positive definite on $\mathcal {X}$ [4, 15]. Furthermore the diagonal-dominance concern reported in Cuturi et al. [15] can be mitigated by appropriate scaling of the local kernel (e.g., the temperature λ) and by avoiding extreme length disparities [4].

To incorporate positional information and reduce cost, [4] introduced *Triangular Global Alignment* (TGA) kernels where the local similarity is modulated by a Toeplitz position kernel $\omega (i,j)=\psi (|i-j|)$ with compact support of width $T \in \mathbb {N}$, i.e. ω(i,j)=0 whenever |i-j|≥T (triangular/band-limited weighting). Here *T* is a natural number user-chosen bandwidth (maximum positional lag) controlling how far from the diagonal matches are allowed. Using such ω within GA yields a p.d. kernel whose computation drops to $O(T\,\min \{n,m\})$.

*What we use in this work.* We employ the standard GA recursion in defined in Equation (1) with a chemically meaningful local kernel at the monomer level (Sect. 2.2), and use cosine (unit-diagonal) normalization of the resulting Gram matrix for GP inference.

$$\hat{K}(A,B)\;=\;\frac{K_{\textrm{GA}}(A,B)}{\sqrt{K_{\textrm{GA}}(A,A)\,K_{\textrm{GA}}(B,B)}}.$$

### Monomer decoupled global alignment kernel

*Representation.* Each cyclic peptide *M* is represented as an ordered sequence of monomer SMILES $(s_1,\ldots ,s_n)$ extracted from CycPeptMPDB [1]. For every monomer we compute a count-based Morgan fingerprint (radius 3, chirality) using RDKit’s GetMorganFingerprint, represented as a sparse, hashed feature vector. This encodes local chemical neighborhoods at the monomer level while preserving sequence order, a crucial factor for cyclic-peptide permeability [3].

*Local chemical kernel.* We use the Tanimoto (Jaccard) similarity between monomer fingerprints as the local kernel,

2 $$\begin{aligned} \kappa _0\!\big (\phi (s),\phi (t)\big )\;=\;\frac{\langle \phi (s),\phi (t)\rangle }{\Vert \phi (s)\Vert _1+\Vert \phi (t)\Vert _1-\langle \phi (s),\phi (t)\rangle }, \end{aligned}$$

The Tanimoto kernel on bit/vector fingerprints is positive definite and widely used in cheminformatics [23, 24]. In particular, for nonnegative fingerprint representations the Tanimoto similarity defines a PSD kernel( [23, 24] for proofs and discussion) In our default model we set $\kappa =\kappa _0$.

*Monomer Decoupled Global Alignment Kernel (MD-GAK)* Let $A=(s_1,\ldots ,s_n)$ and $B=(t_1,\ldots ,t_m)$, two sequences of monomers representing two peptides, respectively. We specialize global alignment to monomer similarities but *decouple* the effect of chemical matches from the effect of gaps. Concretely, let $\kappa (\phi (s_i),\phi (t_j))\in [0,1]$ be the local monomer kernel (Sect. 2.3), and let λ=1 be a gap decay. We define the dynamic program

3 $$\begin{aligned}&M_{0,0}=1,\quad M_{i,0}=M_{0,j}=0,&\nonumber \\&M_{i,j}=\kappa \!\big (\phi (s_i),\phi (t_j)\big )\,M_{i-1,j-1}+\lambda \,M_{i-1,j}+\lambda \,M_{i,j-1},&\end{aligned}$$

and set the kernel value to $K_{\mathrm {MD\text{- }{}GAK}}(A,B):=M_{n,m}$. Finally, by using cosine normalization we scale $K_{\mathrm {MD\text{- }{}GAK}}$ in [0, 1].

$$\hat{K}_{\mathrm {MD\text{- } GAK}}(A,B)\;=\;\frac{K_{\mathrm {MD\text{- } GAK}}(A,B)}{\sqrt{K_{\mathrm {MD\text{- } GAK}}(A,A)\,K_{\mathrm {MD\text{- } GAK}}(B,B)}}.$$

*Why the decoupling helps.* In the canonical GA update

$$M_{i,j} = \kappa \big (\phi (s_i),\phi (t_j)\big )\, \big (M_{i-1,j-1}+M_{i-1,j}+M_{i,j-1}\big ),$$

the local similarity $\kappa (\phi (s_i),\phi (t_j))$ multiplicatively controls all three transitions into (*i*, *j*). A poor local match ($\kappa \approx 0$) drives $M_{i,j}$ close to zero regardless of how good the predecessor states are, so any alignment path that must pass through (*i*, *j*) is heavily downweighted. This can make the kernel overly sensitive to isolated mismatches and encourage reliance on a few high-κ matches.

In contrast, the decoupled MD-GAK recursion in (3) separates the roles of matches and gaps. If $\kappa \big (\phi (s_i),\phi (t_j)\big )=0$, then

$$M_{i,j} = M_{i-1,j} + M_{i,j-1},$$

so the dynamic program can simply bypass the mismatch at (*i*, *j*) through gap steps, with their cost determined solely by $M_{i-1,j}$ and $M_{i,j-1}$. More generally, $\kappa \big (\phi (s_i),\phi (t_j)\big )$ affects only the diagonal (match) transition, while insertions and deletions are controlled independently (by λ=1 in our setting). This encodes the inductive bias that chemical similarity belongs to matches, and penalties belong to gaps.

To illustrate this effect, consider two monomer sequences A=[a,b,c] and D=[a,z,b,c], where *z* is an inserted monomer that is chemically dissimilar to *b*. The desired alignment is a↔a, $\textrm{gap} \leftrightarrow z$, b↔b, and c↔c, thereby recovering the conserved ordered motif [*a*, *b*, *c*]. In the canonical GAK update, paths passing through cells associated with the poor local comparison *b* versus *z* are down-weighted because the same local similarity term controls all incoming transitions. In MD-GAK, the poor Tanimoto similarity between *b* and *z* penalizes only the diagonal substitution b↔z, while the gap transition that skips *z* is governed separately by the gap parameter. Thus, MD-GAK can favor a gap-based alignment around the inserted monomer rather than over-penalizing the entire path due to a local chemical mismatch.

A practical consequence is improved robustness: MD-GAK is less sensitive to single mismatches, and long gap runs do not accumulate large products of local similarities. The runtime remains *O*(*nm*) per pair. In the next section, we show that the decoupled global alignment still defines a valid positive semidefinite kernel.

#### Theorem 1

(Positive semidefiniteness of the Decoupled GA kernel) *Let* $\mathcal {X}$* be the set of monomers and let* $k:\mathcal {X}\times \mathcal {X}\rightarrow \mathbb {R}_{\ge 0}$* be a positive semidefinite (PSD) local kernel (e.g., Tanimoto on Morgan fingerprints). Fix *λ=1.* For sequences *$A=(s_1,\dots ,s_n)$* and *$B=(t_1,\dots ,t_m)$, define the dynamic program

$$M_{0,0}=1,\qquad M_{i,0}=M_{0,j}=0,\qquad M_{i,j}=k(s_i,t_j)\,M_{i-1,j-1}+M_{i-1,j}+M_{i,j-1},$$

*and set *$K(A,B):=M_{n,m}$ *. Then K is a PSD kernel on the space of finite monomer sequences*.

The proof is provided in Appendix.1.

*Position-aware MD-GAK (PMD-GAK).* To encode positional priors and obtain banded computation, we modulate the local similarity by (i) a soft-match transform and (ii) a compactly supported Toeplitz position kernel (the *triangular* window) as in Triangular GA [4]. We take the Tanimoto kernel $\kappa _0$ on Morgan fingerprints introduced above as a base monomer kernel, which satisfies $\kappa _0\in [0,1]$, and define the distance-like score $\varphi =1-\kappa _0$. For β>0 we use the soft local kernel

$$\kappa _\beta \!\big (\phi (s_i),\phi (t_j)\big ) \;=\; \exp \!\Big (-\beta \,\big [1-\kappa _0\!\big (\phi (s_i),\phi (t_j)\big )\big ]\Big ).$$

Let the triangular Toeplitz position kernel of bandwidth $T\in \mathbb {N}$ be

$$\omega _T(i,j)\;=\;\max \!\Big \{0,\;1-\frac{|i-j|}{T}\Big \},\qquad \omega _T(i,j)=0\ \text {if }|i-j|>T.$$

**Fig. 2:**
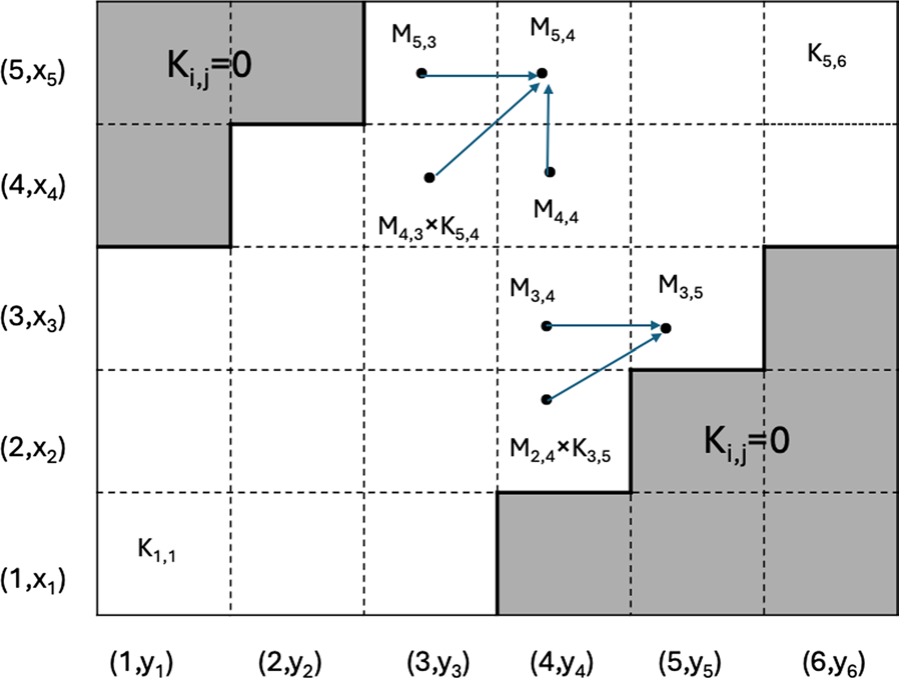
Illustration of the PMD-GAK dynamic program with a compactly supported Toeplitz position kernel $\omega _T(i,j)=\psi (|i-j|)$ of bandwidth T=3. Gray cells indicate entries where $\omega _T(i,j)=0$ (hence $\kappa _T(i,j)=0$), so $M_{i,j}$ does not need to be updated. For white cells inside the band, $M_{i,j}$ is computed from its three predecessors according to $M_{i,j}=\kappa _T(i,j)M_{i-1,j-1}+M_{i-1,j}+M_{i,j-1}$ (arrows). Because $\omega _T(i,j)$ depends only on the index difference |i-j| (Toeplitz structure), the nonzero entries form a diagonal band around the main diagonal

Our position-aware local kernel is $\kappa _T(i,j)=\omega _T(i,j)\,\kappa _\beta (\phi (s_i),\phi (t_j))$, and the DP becomes

4 $$\begin{aligned} M_{0,0}=1,\; M_{i,0}=M_{0,j}=0,\qquad M_{i,j}=\kappa _T(i,j)\,M_{i-1,j-1}+M_{i-1,j}+M_{i,j-1}, \end{aligned}$$

with $K_{\mathrm {PMD\text{- }{}GAK}}(A,B)=M_{n,m}$ and optional cosine normalization ${\hat{K}}_{\mathrm {PMD\text{- }{}GAK}}(A,B)=M_{n,m}$. Because the triangular window $\omega _T$ is Toeplitz and compactly supported, the computation is restricted to the diagonal band |i-j|≤T, visiting $O\!\big (T(n{+}m)\big )$ cells in practice (Fig. 2). Under the same sufficient conditions used for global-alignment kernels—namely, a suitably transformed positive-definite local kernel and a positive-definite positional kernel—the position-weighted similarity remains positive definite.

#### Theorem 2

(Positive semidefiniteness of the Position-aware Decoupled GA kernel)* Let* $\mathcal {X}$* be the set of monomers and let *$\kappa _0:\mathcal {X}\times \mathcal {X}\rightarrow [0,1]$* be a positive semidefinite (PSD) local kernel (e.g., Tanimoto on Morgan fingerprints). Define the distance-like score *$\varphi (x,y)=1-\kappa _0(x,y)$* and, for *β>0*, the soft local kernel*

$$\kappa _\beta \!\big (\phi (s_i),\phi (t_j)\big ) \;=\; \exp \!\Big (-\beta \,\big [1-\kappa _0\!\big (\phi (s_i),\phi (t_j)\big )\big ]\Big ).$$

*Let the triangular Toeplitz position kernel of bandwidth* $T\in \mathbb {N}$* be*

$$\omega _T(i,j)\;=\;\max \!\Big \{0,\;1-\frac{|i-j|}{T}\Big \},\qquad \omega _T(i,j)=0\ \text {if }|i-j|>T.$$

*Define the position-aware local kernel*

$$\kappa _T(i,j)\;=\;\omega _T(i,j)\,\kappa _\beta \big (\phi (s_i),\phi (t_j)\big ).$$

*For sequences *$A=(s_1,\dots ,s_n)$ and $B=(t_1,\dots ,t_m)$*, define the dynamic program*

$$M_{0,0}=1,\qquad M_{i,0}=M_{0,j}=0,\qquad M_{i,j}=\kappa _T(i,j)\,M_{i-1,j-1}+M_{i-1,j}+M_{i,j-1},$$

*and set* $K_{\mathrm {PMD\text{- }{}GAK}}(A,B)=M_{n,m}$ *. Then* $K_T$ *is a PSD kernel on the space of finite monomer sequences.*

The proof is provided in Appendix.2.

*Relation to graph/OT kernels.* MD-GAK/PMD-GAK align sequences of chemically meaningful monomers, whereas recent OT-based graph kernels used with GPs operate directly on molecular (atom–bond) graphs. In particular, the Wasserstein Weisfeiler–Lehman (WWL) kernel and its Sliced-Wasserstein variant (SWWL) embed node attributes via a (continuous) WL scheme and then compare the resulting distributions with (sliced) Wasserstein distances, yielding positive-definite graph kernels well-suited to GP regression on large graphs [5, 25]. For cyclic peptides—where local residue chemistry and backbone ordering both matter—monomer-aware alignment provides a complementary inductive bias: local chemistry shapes match scores through $\kappa _0$, while ordering and allowable warps are handled by the alignment DP (optionally modulated by the positional window $\omega _T$). By contrast, WWL/SWWL aggregate over distributions of WL node embeddings and do not *explicitly* align monomer sequences or enforce a global cyclic order (unless such order is encoded as graph attributes or special edge directions) [5, 25].

### Gaussian processes with molecular fingerprint kernels for peptides

In small-molecule chemoinformatics, Gaussian processes (GPs) commonly use the *Tanimoto* (Jaccard) kernel on binary molecular fingerprints (e.g., ECFP/Morgan), and have been deployed for regression, classification, and Bayesian optimisation [24, 26–28]. In protein sequence design, recent BO work has also explored GP surrogates with either string or fingerprint-style kernels defined on sequence encodings [29]. However, to our knowledge, applying a molecular fingerprint kernel GP directly to model cyclic-peptide permeability, and comparing it head-to-head against peptide-specific alignment kernels (GAK/MD-GAK/PMD-GAK) within the same evaluation protocol, has not been reported prior to this work.

*Fingerprint representation and kernel.* We used the same similarity directly as described in Eq. 2 at the whole-peptide level. Tanimoto kernels on bit vectors are positive (semi)definite and standard in GP modelling for molecules [24, 27]. This yields our GP with TAN_sim model.

*Convex kernel combination.* To probe complementarity between our decoupled global alignment and fingerprint similarity, we also use a convex combination

5 $$\begin{aligned} K_{\textrm{mix}} \;=\; \alpha \,K_{\mathrm {MD-GAK}} \;+\; (1-\alpha )\,K_{\textrm{TAN}}, \qquad \alpha \in [0,1], \end{aligned}$$

with α selected by inner validation. This preserves positive definiteness and lets the GP interpolate between alignment-aware and substructure-aware inductive biases.

Fingerprint-kernel GPs are well established for small molecules and Bayesian Optimization (BO) (e.g., GAUCHE’s Tanimoto kernels [26]; FlowMO’s GPU Tanimoto GP [27]; ordinal-chemistry GP using Tanimoto distances [28]; random-feature approximations for Tanimoto [24]).

### Dataset and data preparation

We use the CycPeptMPDB [1], which compiles permeability measurements for ∼7,334 cyclic peptides (sequence lengths 2–15) drawn from dozens of primary sources and multiple assays, including PAMPA, Caco-2, MDCK and RRCK. Permeability values are reported on a logarithmic scale and clipped to [-10,-4] in CycPeptMPDB; peptides with P≥-6 are generally regarded as cell-permeable [1, 3]. We evaluate our models in two complementary settings detailed below.

*Setting A: applicability-domain–aware splits* Following the data-handling principles and splitting strategies highlighted by Geylan et al. [2], we first extract the SMILES, monomer sequences and PAMPA values for all peptides with available PAMPA in CycPeptMPDB (7,298 entries after initial parsing). To mitigate data leakage from near-identical structures, we group duplicates by canonical SMILES (chirality retained) using Morgan fingerprint (radius 3); for each group we average reported PAMPA values to obtain a single label, yielding 7,221 unique peptides and 276 unique monomers overall. Averaging duplicates is a common choice Liu et al. [3].

*Nested cross-validation.* 5 outer folds (80% train / 20% test per outer fold) and, within each outer training split, a 5-fold inner CV for model selection. Using the P≥-6 threshold, the resulting class counts are 4,801 non-permeable and 2,420 permeable peptides. In line with Geylan et al. [2, Experiment 7], we consider two stratification schemes when constructing folds/splits:

(i) *Label-stratified* (baseline): stratify by the binary permeability label (P≥-6 positive) to preserve class balance across folds. (ii) *Canonical-group–stratified*: stratify by canonical groups, ensuring all members of a group reside in the same fold to curb leakage from highly similar peptides. For this setting, only PAMPA measurements are used (to reduce inter-assay variability), to avoid source-driven leakage and to define a clear applicability domain. We report ACC, F1, ROC–AUC, and Expected Calibration Error (ECE) with $M{=}30$ equal-width bins to measure miscalibration gap between predictive confidence and accuracy [30–33]:

$$\mathbb {E}\big [\Pr (Y=\hat{Y}\mid \hat{P}=p)-p\big ],$$

*Setting B: length-focused PAMPA subset and scaffold splits * To mirror the benchmark protocol of Liu et al. [3], we narrow the chemical space to the sequence lengths with sufficient coverage (6, 7 and 10) and exclude non-PAMPA assays. This yields 5,758 peptides plus 68 peptides with duplicate PAMPA measurements; these duplicates are retained as independent samples but *always* allocated to the training set during splitting to prevent leakage, giving a working subset of 5,826 samples [33]. We evaluate two splitting strategies: (i) *Random split*: 8:1:1 into train/validation/test (repeated 10 times with different seeds), resulting in 4,674/576/576 samples per split. (ii) *Scaffold split*: generate Murcko scaffolds with RDKit (ignoring chirality), sort scaffolds by frequency, assign the most frequent to training and the most diverse to test within each sequence-length bucket, then merge to an overall 8:1:1 split (4,721/554/551) [3, Methods]. Binary labels use the same threshold P≥-6 (1 = permeable, 0 = non-permeable). As in Liu et al. [3], this split design probes generalization both under i.i.d. (*random*) and distribution-shifted (*scaffold*) regimes. We report ROC-AUC Score, aligned with the benchmarks.

### Model implementation

*Baseline models* We used 2048-bit Morgan fingerprints with radius 3 and total counts for representing peptides smiles to the models which is a robust choice for peptide representations. We trained GP with GAK [4], Random Forest [34], XGBoost [35], fine-tuned ChemBerta [12], and PeptideCLM [13] as baseline models for the applicability domain-aware setting. We tuned models in inner loops validation split and used the best hyper parameters on validation set and reported the model performance on the test results. in setting B, We compared the results on pre-specified benchmarks in [3] including RF [34], SVM [36], AttentiveFP [17], DMPNN [18], GAT [37], GCN [38], MPNN [16], PAGTN [39], RNN [40], LSTM [41], GRU [42], ChemCeption [43], ImageMol [44], Multi-CycGT [45] and MUCoCP [46].

## Results

### Setting A: applicability-domain–aware splits

We evaluate two leakage-controlled protocols: (i) label-stratified splits and (ii) canonical-group–stratified splits that remove near-duplicates. Tables 1 and 2 report results for alignment-aware GPs (GAK/MD-GAK/PMD-GAK) versus strong vector baselines (XGBoost, RF) and transformer language-model baselines (ChemBERTa and PeptideCLM). Tables 4 and 5 extend the comparison to fingerprint kernels: a GP with Tanimoto similarity (TAN_sim) and its convex combination with DGAK. All metrics are reported as mean ± s.e.m. over outer folds; *all values are scaled by 100* for readability.

*Label-stratified split (alignment vs. vector baselines).* Under label stratification (Table 1), the GP with MD-GAK achieves the best threshold metrics—ACC (83.0 ± 0.5) and F1 (73.7 ± 0.9)—while PMD-GAK attains the lowest **Brier** (13.83 ± 0.56) and the best ECE among GPs (9.94 ± 1.85), indicating stronger calibration. Tree ensembles display competitive calibration (ECE 8.88/9.07) but *lower discrimination* (ROC–AUC 86.3/85.7) relative to alignment GPs.

**Table 1:** Label-stratified split (values ×100)

Model	ACC	F1	ROC-AUC	Brier score	ECE
GP with GAK kernel	81.1 ± 1.4	69.9 ± 2.9	86.1 ± 1.3	15.93 ± 0.52	15.16 ± 1.69
**GP with MD-GAK kernel**	**83.0** ± **0.5**	**73.7** ± **0.9**	87.8 ± 0.7	14.30 ± 0.46	12.31 ± 1.61
GP with PMD-GAK kernel	82.6 ± 0.7	73.2 ± 0.9	87.6 ± 0.5	**13.83** ± **0.56**	9.94 ± 1.85
XGBoost	78.4 ± 1.2	70.8 ± 1.1	86.3 ± 0.6	14.90 ± 0.51	8.88 ± 0.66
RF	78.3 ± 1.2	70.1 ± 1.2	85.7 ± 0.8	15.07 ± 0.57	9.07 ± 1.60
ChemBERTa	78.6 ± 0.8	69.0 ± 0.5	84.4 ± 0.9	16.03 ± 0.72	11.38 ± 0.92
PeptideCLM	75.9±1.7	67.2±1.7	82.4±1.8	16.7±1.0	**7.8** ± **2.3**

Alignment-aware GPs improve threshold metrics and calibration over vector baselines while maintaining strong discrimination

*Canonical-group–stratified split (alignment vs. vector baselines).* When stratifying by canonical groups (Table 2), the task is harder across the board, consistent with reduced leakage from near-duplicates. PMD-GAK attains the best ACC/F1 (80.3/68.2), the lowest Brier (15.42), and the strongest ROC–AUC among GPs (84.8), while maintaining competitive ECE. Tree ensembles retain the best ECE (XGBoost 9.42; RF 10.52) but trail the best GP in ROC–AUC.

**Table 2:** Canonical-group–stratified split (values ×100)

Model	ACC	F1	ROC-AUC	Brier score	ECE
GP with GAK kernel	79.0 ± 1.4	65.3 ± 4.0	83.0 ± 1.1	20.53 ± 0.45	22.29 ± 1.33
GP with MD-GAK kernel	80.1 ± 1.4	67.8 ± 4.4	84.7 ± 1.0	15.89 ± 0.61	13.35 ± 1.52
**GP with PMD-GAK kernel**	**80.3** ± **1.7**	**68.2** ± **3.8**	**84.8** ± **1.4**	**15.42** ± **0.64**	11.94 ± 1.93
XGBoost	76.4 ± 2.9	64.6 ± 8.5	83.9 ± 1.7	15.73 ± 1.02	9.42 ± 1.48
RF	75.2 ± 2.9	62.4 ± 6.4	81.8 ± 2.4	16.59 ± 1.32	10.52 ± 2.82
ChemBERTa	75.3 ± 1.9	62.1 ± 4.1	80.0 ± 0.9	18.43 ± 0.97	13.78 ± 0.82
PeptideCLM	73.8±2.2	63.8±3.1	78.8±1.4	17.7±0.7	**7.2** ± **1.2**

PMD-GAK provides the best overall balance of discrimination and calibration under the harder split

**Fig. 3:**
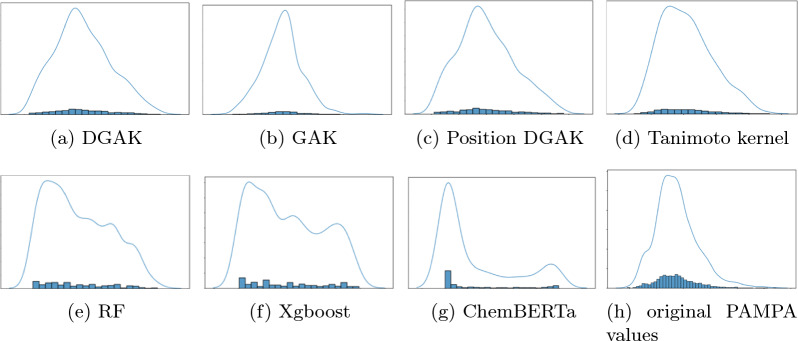
Predicted probability distributions (outer test, canonical-group split). The x-axis shows the predicted probabilities, and the y-axis shows their estimated density of these probabilities on the test set. Kernel-based GP models produce score histograms that closely track the empirical distribution of PAMPA values in the dataset, whereas RF, XGBoost, and ChemBERTa yield noticeably different score profiles. This alignment is consistent with their stronger calibration (lower Brier/ECE; Tables 1, 2) and suggests that the monomer-aware kernels capture permeability-relevant sequence/chemical structure more effectively under canonical splitting

*Score distributions.* Figure 3 compares model score histograms on the outer test sets under canonical-group stratification. Kernel GPs track the empirical PAMPA distribution more closely (after rescaling), while RF, XGBoost, and ChemBERTa yield skewed or multi-modal profiles. The alignment-aware inductive bias thus provides both improved discrimination and better-behaved probabilistic outputs under the harder split.

### Setting B: length-focused PAMPA subset (random vs. scaffold)

We further benchmark on a length-focused PAMPA subset containing 6/7/10-mers under two evaluation regimes: a *random* split and a more stringent *scaffold* split. Table 3 reports ROC–AUC (mean ± s.e.m.; values ×100).

The GP with MD-GAK attains the strongest discrimination on both splits, reaching 88.8 ± 0.**2** (random) and 79.8 ± 0.0 (scaffold). Compared to the best reported graph models under the same protocols, MD-GAK outperforms AttentiveFP on the random split (86.2 ± 1.8) and MPNN on the scaffold split (73.4 ± 8.7). Classical vector baselines (RF/SVM) are substantially weaker on both splits.

*Generalization across scaffolds.* Moving from random to scaffold evaluation reduces performance for all methods, as expected when near-duplicates are removed. The MD-GAK GP exhibits a *9.0*-point drop (88.8 → 79.8), which is markedly smaller than the *20.1*-point drop observed for AttentiveFP (86.2 → 66.1), suggesting that the alignment-aware inductive bias confers improved robustness to scaffold shifts. MPNN’s scaffold performance is relatively stable but remains below the GP with MD-GAK.

**Table 3:** Length-focused PAMPA (6/7/10-mers): ROC–AUC under random vs. scaffold splits (values ×100)

Method	Random split: Classification—binary labels	Scaffold split: Classification—binary labels
*Alignment-aware GP and classical ML*		
GP with MD-GAK	88.8±0.2	79.8±0.0
RF	65.1±4.1	55.3±3.4
SVM	59.6±1.8	53.9±0.1
*Graph-based baselines*		
AttentiveFP (best random)	86.2±1.8	66.1±5.4
DMPNN	84.8±3.2	71.6±7.9
GAT	83.9±4.4	65.9±9.6
GCN	77.2±5.5	66.9±10.4
MPNN (best scaffold)	77.2±5.5	73.4±8.7
PAGTN	78.0±6.2	68.2±3.2
*String-based baselines*		
RNN	55.2±8.0	52.5±3.4
LSTM	56.8±15.0	53.1±10.0
GRU	73.1±14.6	61.7±13.8
*Image-based baselines*		
ChemCeption	46.6±5.1	40.3±4.3
ImageMol	80.4±1.9	66.1±3.7

### Adding fingerprint kernels: discrimination vs. calibration

We now introduce peptide-level molecular fingerprints with a Tanimoto kernel GP (TAN_sim) and a convex mixture with DGAK,(Tables 4 and 5). Under label stratification, TAN_sim delivers the highest ROC–AUC (89.0 ± 0.8), while MD-GAK remains best on ACC/F1 and PMD-GAK remains best calibrated (Brier/ECE). Under canonical-group stratification, TAN_sim again yields the top ROC–AUC (86.7 ± 0.5). The convex mixture achieves the best ACC/F1 (81.1/69.9), suggesting complementary inductive biases between alignment and substructure similarity. Overall, fingerprint similarity increases *rank discrimination*, whereas alignment-aware kernels improve *probability calibration*;

**Table 4:** Label-stratified split with fingerprint kernels (values ×100). Tanimoto improves rank discrimination; MD/PMD-GAK retain threshold and calibration advantages

Model	ACC	F1	ROC-AUC	Brier score	ECE
GP with MD-GAK kernel	**83.0** ± **0.5**	**73.7** ± **0.9**	87.8 ± 0.7	14.30 ± 0.46	12.31 ± 1.61
GP with PMD-GAK kernel	82.6 ± 0.7	73.2 ± 0.9	87.6 ± 0.5	**13.83** ± **0.56**	**9.94** ± **1.85**
GP with TAN_sim kernel	82.8 ± 1.0	73.7 ± 1.4	**89.0** ± **0.8**	14.40 ± 0.32	14.42 ± 0.40
GP with convex (DGAK + Tanimoto)	82.8 ± 0.7	73.6 ± 1.1	88.7 ± 0.9	14.44 ± 0.33	14.28 ± 0.27

Bold values indicate the best performance in each column among the compared models: the highest values for ACC, F1, and ROC–AUC, and the lowest values for Brier score and ECE

**Table 5:** Canonical-group–stratified split with fingerprint kernels (values ×100). Tanimoto again maximizes ROC–AUC; the convex mixture recovers the best ACC/F1 under the harder split

Model	ACC	F1	ROC-AUC	Brier score	ECE
GP with MD-GAK kernel	80.1 ± 1.4	67.8 ± 4.4	84.7 ± 1.0	15.89 ± 0.61	13.35 ± 1.52
GP with PMD-GAK kernel	80.3 ± 1.7	68.2 ± 3.8	84.8 ± 1.4	**15.42** ± **0.64**	**11.94** ± **1.93**
GP with TAN_sim kernel	80.9 ± 1.5	69.3 ± 3.1	**86.7** ± **0.5**	15.57 ± 0.79	13.96 ± 4.42
GP with convex (DGAK + Tanimoto)	**81.1** ± **1.4**	**69.9** ± **2.9**	86.1 ± 1.3	15.93 ± 0.52	15.16 ± 1.69

Bold values indicate the best performance in each column among the compared models: the highest values for ACC, F1, and ROC–AUC, and the lowest values for Brier score and ECE

Finally, we assess peptide-level molecular fingerprints on the length-focused subset via a GP with Tanimoto similarity (TAN_sim) and a convex combination with MD-GAK. As shown in Table 6, TAN_sim achieves the highest ROC–AUC on both splits (random: 0.897 ± 0.002; scaffold: 0.804 ± 0.000), narrowly ahead of the MD-GAK GP. The convex mixture (*MD-GAK + Tanimoto*) matches the top ROC–AUC.

The differences among GP kernels in Tables 4 and 5 should be interpreted cautiously. For fixed folds, preprocessing, and selected hyperparameters, the GP models are deterministic; the reported variability therefore reflects variation across data splits rather than stochastic retraining noise. Several differences in ACC, F1, and ROC–AUC are small relative to this fold-to-fold variability, indicating that the GP kernels are not always statistically distinguishable on discrimination metrics alone. We therefore view MD-GAK and PMD-GAK not as uniformly dominant over all GP kernel choices, but as competitive monomer-aware alignment kernels that provide a peptide-specific inductive bias while maintaining performance comparable to strong whole-molecule Tanimoto kernels.

**Table 6:** Length-focused PAMPA (6/7/10-mers): ROC–AUC under random vs. scaffold splits using fingerprint kernels (raw values, not scaled)

Method	Random split: Classification – binary labels	Scaffold split: Classification – binary labels
AttentiveFP (best random)	86.2±1.8	66.1±5.4
MPNN (best scaffold)	77.2±5.5	73.4±8.7
GP with MD-GAK	88.8±0.2	79.8±0.0
**GP with** TAN_sim	89.7±0.2	80.4±0.0
GP with convex (MD-GAK + Tanimoto)	89.7±0.2	80.4±0.0

### Circular alignment ablation

To assess the effect of the arbitrary linearization of cyclic peptides, we implemented a rotation-invariant circular GAK baseline. Starting from the validated GAK baseline with monomer-level Tanimoto local similarity, we averaged the kernel over all cyclic rotations of both peptide monomer sequences:

$$K_{\textrm{cGAK}}(x,y) = \frac{1}{|x||y|} \sum _{r=0}^{|x|-1} \sum _{s=0}^{|y|-1} K_{\textrm{GAK}}\left( \textrm{rot}_r(x),\textrm{rot}_s(y)\right) .$$

This construction tests all possible choices of the starting residue at the sequence level and preserves positive semidefiniteness because it is an average of positive-semidefinite kernels evaluated on rotated inputs. This circular GAK is a sequence-level rotation-invariant kernel and should be distinguished from a full cactus-graph dynamic program over peptide topology.

Circular GAK produced only marginal changes relative to the linearized GAK baseline (Table 7). Under the label-stratified split, circular GAK changed F1 from 69.9±2.9 to 69.9±0.8, and ROC-AUC from 86.1±1.3 to 86.2±0.5. Under the canonical-group split, circular GAK changed F1 from 65.3±4.0 to 65.7±2.3, and ROC-AUC from 83.0±1.1 to 83.1±0.4. These results suggest that, in the present CycPeptMPDB benchmark, the arbitrary choice of sequence origin has little practical effect on the GAK-family kernel performance. Nevertheless, full cactus-graph alignment remains an interesting future direction for lariat, branched, or side-chain-cyclized peptides whose topology cannot be represented by a single cyclic order.

**Table 7:** Circular GAK ablation. Values are reported as mean ± standard error across outer folds and scaled by 100

Kernel	Split	ACC	F1	ROC-AUC	Brier	ECE
GAK	Label-stratified	81.1±1.4	69.9±2.9	86.1±1.3	15.93±0.52	15.16±1.69
Circular GAK	Label-stratified	81.5±0.6	69.9±0.8	86.2±0.5	15.89±0.11	15.13±0.43
GAK	Canonical-group	79.0±1.4	65.3±4.0	83.0±1.1	20.53±0.45	22.29±1.33
Circular GAK	Canonical-group	79.3±0.3	65.7±2.3	83.1±0.4	20.44±0.07	22.41±0.22

### Continuous PAMPA regression

Although the main task is framed as binary permeability classification for screening, PAMPA permeability values are continuous. We therefore added a Gaussian-process regression experiment to evaluate the kernels independently of the classification threshold. We used the same precomputed GAK, MD-GAK, and PMD-GAK Gram matrices and reused the same outer and inner folds as in the label-stratified classification experiment in Table 1 for comparability. Models were trained on the continuous PAMPA values rather than thresholded labels. Hyperparameters were selected within the inner folds by minimizing mean squared error, and the held-out outer folds were evaluated using MSE, MAE, and RMSE. As shown in Table 8, MD-GAK and PMD-GAK gave lower regression errors than the standard GAK baseline across all three metrics, indicating that the monomer-decoupled kernels capture useful continuous permeability structure beyond the binary threshold.

**Table 8:** Continuous PAMPA regression results under the label-stratified split. Values are reported as mean ± standard error across outer folds and scaled by 100. Lower values are better for all metrics

Model	MSE ↓	MAE ↓	RMSE ↓
GP regression with GAK	72.44±4.20	52.79±1.07	84.97±2.43
GP regression with MD-GAK	65.16±5.17	49.09±1.25	80.49±3.06
GP regression with PMD-GAK	65.58±4.30	49.57±1.00	80.82±2.58

## Discussion

This work aims to reconcile two desiderata for cyclic peptides: a better representation that preserves ordered monomer chemistry and a model that remains data-efficient with calibrated uncertainties. To this end, we introduced a monomer–aware global alignment family for GPs (MD-GAK and the position-aware PMD-GAK) and evaluated them under applicability-domain–aware protocols that curb leakage from near-duplicates. On CycPeptMPDB, alignment-aware GPs consistently improve discrimination over strong vector and language-model baselines, while PMD-GAK yields the best calibration among GPs (lower Brier/ECE). Under the stricter canonical-group split, the advantage of alignment is amplified (Table 2), and on a length-focused PAMPA subset our GP exceeds, leading graph baselines (Table 5). These findings are robust across splits and metrics, with uncertainty estimates that are well-behaved in the harder setting (Fig. 2).

Although ROC–AUC remains a useful ranking metric, permeability datasets are label-imbalanced and downstream decisions hinge on operating at a single threshold. In such settings, precision–recall criteria—and in particular the $F_{1}$ score (the harmonic mean of precision and recall)—are more informative than ROC–AUC because ROC can present an overly optimistic picture under class skew [47, 48]. For this reason, we focus our head-to-head comparison on F1 in Tables 1,2, under label stratification, MD-GAK achieves the best F1 (73.7 ± 0.9), and under canonical stratification, PMD-GAK leads (68.2 ± 3.8), while retaining competitive AUCs and improved calibration (lowest Brier/ECE among GPs). This strengthens the practical relevance of alignment kernels for actionable screening, where precision/recall trade-offs matter at deployment time.

*Harder tasks magnify the benefit of alignment.* When we move from label stratification to canonical-group stratification—explicitly removing near-duplicate peptides—performance drops for all methods, but the relative improvement of MD-GAK/PMD-GAK over baselines is stronger. This is consistent with the hypothesis that alignment captures permeability-relevant order and context beyond bag-of-substructures; once leakage is reduced, these inductive biases become more valuable (Table 1).

*Calibration matters.* Well-calibrated probabilities enable principled triage in low-data discovery loops. We observe systematically lower Brier/ECE for PMD-GAK relative to other GPs, and tree ensembles remain well calibrated but less discriminative. This mirrors broader evidence that modern ML methods can be poorly calibrated without post-hoc correction [31]. In screening settings with limited assays, these calibrated GP posteriors are advantageous for ranking and for uncertainty-aware decision making.

*Tanimoto at the* *monomer* *level connects peptides to the small-molecules.* A key design choice was to score monomer matches with a Tanimoto kernel on Morgan fingerprints inside the alignment DP. This shows that Tanimoto is effective at the monomer level for peptides, achieving strong performance. Importantly, this choice bridges peptide modeling with the mature small-molecule toolkit: decades of kernels, scalable approximations, and software are immediately compatible. For instance, scalable Tanimoto approximations via random features [24] and comprehensive GP tooling for chemistry (GAUCHE) [26] can be dropped in without architectural changes. More broadly, our decoupled setup invites learned monomer encoders from chemical language models (e.g., SMILES Transformers, ChemBERTa, MolT5) to provide richer local descriptors within the same GA framework [12, 49, 50]. In other words, by proving that "Tanimoto@monomer" is a strong building block, we open a path to upgrade the local kernel κ with powerful, pre-trained small-molecule representations as they continue to improve.

*Complementarity of alignment and substructure similarity.* Across settings, the Tanimoto fingerprint GP (TAN_sim) consistently maximizes *rank discrimination* (ROC–AUC), whereas alignment variants (MD-/PMD-GAK) dominate threshold metrics (ACC/F1) and calibration (Brier/ECE). A simple convex kernel (DGAK + Tanimoto) recovers much of both: it matches the strongest AUCs while achieving the best ACC/F1 under the canonical split (Tables 4–5). These results support a clear division of labor: local substructures captured by fingerprints and ordered monomer context captured by alignment can encode distinct, complementary signals. In practice, when both high precision/recall and reliable probabilities are required, the convex combination provides a strong, low-complexity default.

*Computational cost.* A practical consideration for MD-GAK and PMD-GAK is computational cost. For two peptides with monomer-sequence lengths $L_i$ and $L_j$, evaluating the kernel requires filling a dynamic-programming table of size $L_i \times L_j$, giving a pairwise cost of $O(L_iL_j)$. For a training set of N peptides with average length $\bar{L}$, constructing the full training kernel matrix therefore costs $O(N^2\bar{L}^2)$ and requires $O(N^2)$ memory. Exact GP training then adds the standard $O(N^3)$ cost for Cholesky factorization or inversion of the N×N kernel matrix. For a test set of M peptides, constructing the test–training kernel matrix costs $O(MN\bar{L}_{\textrm{test}}\bar{L}_{\textrm{train}})$. In the present CycPeptMPDB setting, peptide lengths are short and the number of training peptides is on the order of a few thousand, so exact GP inference remains feasible. For substantially larger datasets, the dominant bottlenecks would be $O(N^2)$ kernel storage and $O(N^3)$ exact-GP factorization, for which sparse or inducing-point GP approximations and low-rank kernel approximations would be natural extensions.

*Limitations and future work.* First, we deliberately avoided heavy hyperparameter search; the convex weight λ and the positional bandwidth in PMD-GAK were set conservatively. A more systematic selection could further improve threshold metrics per split. Relatedly, moving from single- to multi-task settings (e.g., PAMPA and Caco-2) may benefit from shared alignment but assay-specific calibration layers.

Second, our local chemistry kernel κ used a single family (Tanimoto on Morgan). While effective, bit-vector fingerprints may underrepresent stereochemistry and conformational effects [11, 51]. A key advantage of the monomer-decoupled formulation is that κ is modular: it need not be restricted to Morgan/Tanimoto similarity. Future work can (i) upgrade κ with richer small-molecule encoders from chemical language models or molecular foundation models, such as SMILES Transformer, ChemBERTa, or MolT5, while retaining the same global-alignment scaffold, or (ii) adopt scalable approximations of Tanimoto via random features when data grow [12, 24, 49, 50]. This modularity is particularly useful for cyclic-peptide permeability modeling because labeled peptide datasets remain comparatively limited, whereas small-molecule resources are much larger, including PubChem-scale corpora. Thus, learned monomer-level kernels or embedding similarities pretrained on large small-molecule datasets could provide a natural route for transferring information from data-rich small-molecule chemistry into peptide-level GP alignment models.

Third, scalability: exact GP inference is $\mathcal {O}(N^{3})$ in the number of training peptides. Although our datasets are moderate, larger campaigns will require sparse/inducing-point GPs or Nyström-style approximations [52]. These are orthogonal to our kernel design and can be combined with MD-/PMD-GAK and fingerprint kernels without changing the modelling interface.

Fourth, structure granularity. Our approach is *sequence-first*: atom-level arrangements within each monomer and macrocycle are only accessed through the local kernel κ. Graph GP kernels that capture distributional node/edge structure—for instance, (Wasserstein) Weisfeiler–Lehman variants or topological kernels based on sliced Wasserstein distances—could complement our alignment bias and enable hybrid sequence–graph GPs for macrocycles [5, 25]. Exploring such hybrids, especially for noncanonical residues and bridged rings, is a promising direction.

Finally, while our results already outperform strong graph baselines on the length-focused subset, more systematic ablations on stereochemistry handling and macrocycle ring closures will clarify where alignment contributes most.

## Conclusion

We introduced monomer-aware global alignment kernels for Gaussian processes (MD-GAK and position-aware PMD-GAK) and showed that they deliver strong discrimination, better calibration, and higher **F1**—especially under the harder canonical-group split where *alignment matters more*. Complementing alignment with peptide-level molecular fingerprints, a Tanimoto GP (TAN_sim) maximized AUC, while a simple convex mixture with MD-GAK recovered both top AUC and improved ACC/F1, confirming the complementarity of local substructures and ordered monomer context. On a length-focused PAMPA subset, our GPs surpassed strong graph baselines and generalized better across scaffolds. Practically, demonstrating that Tanimoto works at the monomer level creates a clean interface to the small-molecule ecosystem (classical kernels, scalable random-feature approximations, chemical LLMs) without changing the alignment scaffold, opening an immediate path to richer local chemistry encoders as data scale grows.

## Supplementary Information

Supplementary material.

## Data Availability

The data used to train the cell-permeability predictive models for cyclic peptides are publicly available in CycPeptMPDB: http://cycpeptmpdb.com/download/.

## Appendix A Proof

### Proof of positive semidefiniteness of the Decoupled GA kernel

Let $s=(s_1,\ldots ,s_n)$ and $t=(t_1,\ldots ,t_m)$ be two sequences of lengths *n* and *m*, respectively, where $s_i, t_j \in \mathcal {X}$. Let $k:\mathcal {X}\times \mathcal {X}\rightarrow \mathbb {R}$ be a valid kernel that compares elements in the sequences. We define as $M\in \mathbb {R}^{(n+1) \times (m+1)}$ the follow matrix

$$M(i,j) = k(s_i, t_j)M(i-1,j-1) + M(i-1,j) + M(i, j-1)$$

with initial conditions M(0,0)=1, M(i,0)=0, and M(0,j)=0. We want to prove that *M*(*m*, *n*) is valid kernel that compares *s* and *t*, i.e. K(s,t)=M(m,n).

A step is one of three moves on the integer lattice: R=(1,0), U=(0,1), and D=(1,1), and a monotone path π from (0, 0) to (*i*, *j*) is a finite sequence of steps that transforms (0, 0) to (*i*, *j*). For each π we collect the set of indices corresponding to diagonal *D* steps.

$$D(\pi ) = \{\, (i_1, j_1),\ldots ,(i_r, j_r), \, i_1<\cdots<i_r, \, j_1< \cdots <j_r \, \}.$$

*Example.* If we consider π as follows

$$\pi :\quad (0,0) \xrightarrow {\textrm{R}} (1,0) \xrightarrow {\textrm{D}} (2,1) \xrightarrow {\textrm{U}} (2,2) \xrightarrow {\textrm{D}} (3,3),$$

$$D(\pi ) = \{ (2,1),(3,3)\}$$.

We define the weight *w* of a path

$$w(\pi ) = \prod _{(i,j) \in D(\pi )} k(s_i,t_j).$$

Note if π has no diagonals, the product is empty and equals to 1. Finally we define the set of all the possible monotonic path from (0, 0) to (*i*, *j*) as $\mathcal {P}(i,j)$.

### Lemma 1

*For each* i,j≥0,* we want to prove the following*

$$M(i,j) = \sum _{(i,j)\in \mathcal {P}(i,j)} w(\pi )$$

*Base case.* For i=j=0, $\mathcal {P}(i,j) = \emptyset .$, so w(∅)=1 by definition and follows that M(0,0)=1, which confirms the inital condition on *M*(0, 0).

*Indution step.* We assume the equality holds for $i^{\prime },j^{\prime }$ such that $i^{\prime }+j^{\prime }<i+j$. Let us know partition $$\mathcal {P}(i,j)$$ as follows

- Paths whose last step is *R* (right) are bijection with $\mathcal {P}(i-1,j)$. It is sufficient to add a *R* step to $\mathcal {P}(i-1,j)$ to get $\mathcal {P}(i,j)$. Note that the bijections holds because w(π) depends only on coordinates on D(π). The right move *R* cannot be in D(π).
- Paths whose last step is *U* (up) are bijection with $\mathcal {P}(i,j-1)$.
- Paths whose last step is *D* (diagonal) are $\pi = \pi ' \circ D$.

Putting all together we have

6 $$\begin{aligned}&\sum _{\pi \in \mathcal {P} (i,j)}w(\pi ) \end{aligned}$$

7 $$\begin{aligned}=&\sum _{\pi ' \in \mathcal {P} (i-1,j)}w(\pi ') + \sum _{\pi ' \in \mathcal {P} (i,j-1)}w(\pi ') + w(\{(i,j)\})\sum _{\pi ' \in \mathcal {P} (i-1,j-1)}w(\pi ') \end{aligned}$$

8 $$\begin{aligned}=&\sum _{\pi ' \in \mathcal {P} (i-1,j)}w(\pi ') + \sum _{\pi ' \in \mathcal {P} (i,j-1)}w(\pi ') + k(s_i,t_j)\sum _{\pi ' \in \mathcal {P} (i-1,j-1)}w(\pi '). \end{aligned}$$

By induction hypothesis since i-1+j<i+j, i+j-1<i+j, and i-1+j-1<i+j, we have that

$$\sum _{\pi \in \mathcal {P} (i,j)}w(\pi ) = M(i-1,j) + M(i,j-1)+k(s_i,t_j)M(i-1,j-1).$$

Note that the paths that start with a right or up move lead to prefixes visiting (*i*, 0) or (0, *j*) with positive index; those prefixes have been given M(i,0)=0 and M(0,j)=0 The induction sums include these cases but the recurrence’s base values ensure they contribute 0, which is consistent with the combinatorial splitting above.

### Observation 1

Since *k* is valid kernel on sequence elements, it must exist a feature map ϕ that maps $\mathcal {X}$ into a feature vector in $\mathbb {R}^d$, i.e.

$$k(s_i, t_j) = \langle \phi (s_i),\phi (t_j)\rangle$$

Let us take now a diagonal path $\pi \in D(\pi )$, where $\pi = \{ (i_1,j_1),\ldots ,(i_r, j_r) \}.$, we define the feature map on $\Phi _{\pi }$ for *s* and *t* as

9 $$\begin{aligned} \Phi _{\pi }(s) = \phi (s_{i_1}) \otimes \phi (s_{i_2}) \otimes \cdots \otimes \phi (s_{i_r}) \end{aligned}$$

10 $$\begin{aligned} \Phi _{\pi }(t) = \phi (t_{i_1}) \otimes \phi (s_{i_2}) \otimes \cdots \otimes \phi (s_{j_r}). \end{aligned}$$

The scalar product between $\Phi _{\pi }(s)$ and $\Phi _{\pi }(t)$ is (by using the properties on Kronecker product ⊗)

$$\langle \Phi _{\pi }(s), \Phi _{\pi }(t) \rangle =\prod _{k=1}^r k(s_{i_k},t_{j_k}) = w(\pi )$$

## Final proof

Let us now construct the feature vector Ψ considering all the paths in $\mathcal {P}(m,n)$ as

$$\Psi (s):= \bigoplus _{\pi \in \mathcal {P}(m,n)} \Phi _{\pi }(s).$$

Similarly, we can construct Ψ(t) and their scalar product is

$$\begin{aligned} \langle \Psi (s),\Psi (t)\rangle = \langle \bigoplus _{\pi \in \mathcal {P}(m,n)} \Phi _{\pi }(s),\bigoplus _{\pi \in \mathcal {P}(m,n)} \Phi _{\pi }(t)\rangle = \sum _{\pi \in \mathcal {P}(m,n)} \langle \Phi _{\pi }(s),\Phi _{\pi }(t)\rangle = \\ \sum _{\pi \in \mathcal {P}(m,n)}w(\pi ) = M(m,n), \end{aligned}$$

which concludes the proof. Since we express *M*(*m*, *n*) as the scalar product of two feature vectors, this is always a valid kernel.

### Proof of positive semidefiniteness of the position-aware decoupled GA kernel

We reuse the notation of Theorem 1. Recall that *K*(*A*, *B*) in Theorem 1 is PSD for *any* PSD local kernel $k:\mathcal {X}\times \mathcal {X}\rightarrow \mathbb {R}_{\ge 0}$. To prove Theorem 2, it therefore is enough to show that the position-aware local kernel

$$\kappa _T(i,j)\;=\;\omega _T(i,j)\,\kappa _\beta \big (\phi (s_i),\phi (t_j)\big )$$

is PSD on the extended alphabet of position–monomer pairs. We proceed in three steps.

### Step 1: the soft local kernel $\kappa _\beta$ is PSD

Let $\kappa _0:\mathcal {X}\times \mathcal {X}\rightarrow [0,1]$ be a PSD kernel (e.g. Tanimoto), and define $\varphi (x,y)=1-\kappa _0(x,y)$. For β>0 define

$$\kappa _\beta (x,y) \;=\; \exp \big (-\beta \,\varphi (x,y)\big ) \;=\; \exp \big (-\beta \,[1-\kappa _0(x,y)]\big ).$$

Fix a finite set of monomers $\{x_1,\dots ,x_N\}\subset \mathcal {X}$ and let

$$K_0 \;=\; \big [\kappa _0(x_p,x_q)\big ]_{p,q=1}^N.$$

By assumption $K_0\succeq 0$ and all entries of $K_0$ lie in [0, 1]. The Gram matrix of $\kappa _\beta$ on this set is

$$[K_\beta ]_{pq} \;=\; \kappa _\beta (x_p,x_q) \;=\; \exp \big (-\beta [1-\kappa _0(x_p,x_q)]\big ) \;=\; e^{-\beta }\,\exp \big (\beta \,\kappa _0(x_p,x_q)\big ).$$

Using the power-series expansion of the exponential,

$$\exp (\beta z) = \sum _{m=0}^\infty \frac{\beta ^m}{m!}\,z^m,\qquad z\in [0,1],$$

we obtain the elementwise expansion

$$K_\beta \;=\; e^{-\beta } \sum _{m=0}^\infty \frac{\beta ^m}{m!}\,K_0^{\ m},$$

where $K_0^{m}$ denotes the *m*-th Hadamard (entrywise) power: $(K_0^{m})_{pq} = (K_0)_{pq}^m$.

Because $K_0\succeq 0$ and has nonnegative entries, the Schur product theorem implies that each Hadamard power $K_0^{m}$ is PSD. All coefficients $e^{-\beta }\beta ^m/m!$ are nonnegative, hence $K_\beta$ is a nonnegative linear combination of PSD matrices and is therefore PSD. As this holds for every finite subset, $\kappa _\beta$ is a positive semidefinite kernel on $\mathcal {X}$.

### Step 2: the triangular position kernel $\omega _T$ is PSD

Fix $T\in \mathbb {N}$. We follow Cuturi [4] and use the triangular Toeplitz position kernel on indices $i,j\in \mathbb {Z}$,

$$\omega _T(i,j) \;=\; \max \Big \{0,\,1-\frac{|i-j|}{T}\Big \},\qquad \omega _T(i,j)=0\ \text {if }|i-j|>T.$$

This is the restriction to $\mathbb {Z}$ of the classical triangular kernel on $\mathbb {R}$, which is known to be positive definite (see Gneiting [53]). Cuturi [4, Sec. 4.3] uses the same kernel in the construction of Triangular Global Alignment (TGA) kernels. It follows that $\omega _T$ is a positive semidefinite kernel on positions $i,j\in \mathbb {N}$.

### Step 3: the position-aware local kernel $\kappa _T$ is PSD

Consider the product space of positions and monomers

$$\widetilde{\mathcal {X}} = \mathbb {Z}\times \mathcal {X},$$

and define

$$k_{\textrm{loc}}\big ((i,s),(j,t)\big ):= \omega _T(i,j)\,\kappa _\beta (s,t).$$

Let $\{(i_p,s_p)\}_{p=1}^N \subset \widetilde{\mathcal {X}}$ be arbitrary, and denote $A_{pq} = \omega _T(i_p,i_q)$ and $B_{pq} = \kappa _\beta (s_p,s_q)$. By Steps 1–2, *A* and *B* are PSD Gram matrices. The Gram matrix of $k_{\textrm{loc}}$ on these points is

$$G_{pq} = k_{\textrm{loc}}\big ((i_p,s_p),(i_q,s_q)\big ) = A_{pq} B_{pq} = (A \circ B)_{pq},$$

the Hadamard product of *A* and *B*. By the Schur product theorem, the Hadamard product of two PSD matrices is PSD, hence G⪰0. Therefore $k_{\textrm{loc}}$ is a PSD kernel on $\widetilde{\mathcal {X}}$.

### Step 4: applying the MD-GAK result

Given a monomer sequence $A=(s_1,\dots ,s_n)$, we associate to it the sequence of position–monomer pairs

$$\widetilde{A} = \big ((1,s_1),\dots ,(n,s_n)\big )\in \widetilde{\mathcal {X}}^{*},$$

and similarly for $B=(t_1,\dots ,t_m)$.

The dynamic program in (4) coincides with the MD-GAK recursion of Theorem 1 applied to the sequences $\widetilde{A},\widetilde{B}$ over the alphabet $\widetilde{\mathcal {X}}$, with local kernel $k_{\text {loc}}$. Since $k_{\text {loc}}$ is PSD, Theorem 1 implies that the resulting sequence kernel

$$K_T(A,B) \;=\; M_{n,m}$$

is positive semidefinite on the space of finite monomer sequences. This completes the proof of Theorem 2.

## Code availability

The implementation code is available at: https://github.com/ali-amirahmadii/PEPTAK.
